# Barriers and facilitators to implementing comprehensive sex education in Texas public schools: A qualitative study

**DOI:** 10.1371/journal.pone.0316329

**Published:** 2025-01-02

**Authors:** Lauren Holt, Sarah Janek, Gavin Yamey

**Affiliations:** 1 School of Nursing, Duke University, Durham, NC, United States of America; 2 Global Health Institute, Duke University, Durham, NC, United States of America; The University of Tennessee Health Science, UNITED STATES OF AMERICA

## Abstract

**Introduction:**

In Texas, the adolescent birth rate is higher than the national average and STIs have steadily increased over the last decade. Sex education is not mandated in Texas and the majority of public schools provide an abstinence-based sex education. Comprehensive sexuality education (CSE) programs are widely endorsed by national and global health organizations and research has shown that they are more effective in reducing poor sexual health outcomes than abstinence-based programs. The purpose of this study is to identify barriers and facilitators to implementing CSE at local and state levels in Texas.

**Methods:**

Qualitative study design consisting of ten semi-structured interviews with eleven key informants (n = 11) conducted in 2021.

**Results:**

Three barriers- ideological opposition to CSE, discrimination against LGBTQ+ people, myths and misconceptions about CSE- and two facilitators- champions, collaboration with community stakeholders- to implementing CSE policy were identified.

**Conclusion:**

Study findings provide insight into the opposition faced by sex education advocates, which often stems from myths and misperceptions of CSE content and the stigmatization of sexual and gender minoritized groups. Parents, youth, medical professionals, and academic researchers are instrumental in dispelling sex education myths and misperceptions and engaging with community stakeholders.

## 1. Introduction

Sexual health education provides young people with the knowledge and skills needed to make informed sexual health decisions to prevent sexually transmitted infections (STIs), including HIV, and unwanted pregnancy. In Texas, the adolescent birth rate is 43% higher than the national average and Texas ranks first in repeat births among adolescents at 18% [1, 2]. Meanwhile, chlamydia and gonorrhea rates have increased by 25% and HIV rates by 4% over the last decade among adolescents and young adults between 15 and 24 years [3, 4].

Currently, Texas is one of 22 states that do not mandate sexual health education and one of 33 states that do not require the provided sex education to be medically accurate [3]. Further, if a Texas public school chooses to provide sex education to students, the curriculum must emphasize abstinence (SEICUS, 2021). Although abstinence is the most effective way to prevent pregnancy and STIs, nearly 43% of Texas high school students have engaged in sexual activity at least once and 23% are currently sexually active [5, 6]. Further, among students who are sexually active, 50% report not using a condom the last time they had sex and 20% report not using any pregnancy prevention method [6]. The lack of contraception use among Texas youth combined with elevated STI and unwanted pregnancy rates highlights the need to consider effective sexual health promotion strategies.

Comprehensive sexuality education (CSE) programs are widely endorsed by national and global health organizations and research has shown that they are more effective in reducing poor sexual health outcomes than abstinence-based programs [7–10]. CSE is a medically accurate curriculum that provides age-appropriate information related to the physical, mental, emotional, and social dimensions of human sexuality [11]. CSE is taught from early elementary through high school and consists of seven key topics: consent and health relationships, anatomy and physiology; puberty and adolescent development; gender identity and expression; sexual orientation and identity; sexual health; and interpersonal violence [12]. Compared to abstinence-based programs, CSE is more effective in delaying sexual debut, increasing use of contraception, and reducing STI/HIV and adolescent pregnancy rates [8–10, 13, 14]. Additionally, CSE has been shown to reduce domestic and intimate partner violence, child sexual abuse, and homophobia and homophobic bullying [15].

Although the effectiveness of CSE over abstinence-based programs is well-supported, few Texas schools provide CSE. To understand where and how Texas sex education policy can be improved, it is essential to learn what the current policies and standards are and who decides them.

### 1.1 State legislature

The state legislature plays a powerful role in sex education policy by passing laws related to sex education, included in The Texas Education Code [16]. The Texas State Board of Education (SBOE) must adhere to all state laws when deciding the standards of Texas Essential Knowledge and Skills (TEKS). According to the Texas Education Code [16] all sex education curricula must “present abstinence from sexual activity as the preferred choice of behavior in relationship to all sexual activity for unmarried persons of school age” and “devote more attention to abstinence from sexual activity than to any other behavior”. Moreover, Texas schools must teach condom effectiveness from the perspective of “human use reality rates” as opposed to “theoretical laboratory rates” [17], meaning the condom failure rate provided by schools reflects incorrect or inconsistent condom use. Through this mandate, students may be led to believe condoms are less effective than they actually are when used correctly or consistently.

The Texas Education Code also defines the process that schools must abide by when selecting sex education curriculum and proposing curriculum changes. Prior to November 2020, all students were automatically enrolled in sex education unless their parents signed a permission slip prohibiting their child from attending [18]. In November 2020, Texas became one of five states to enforce a new policy requiring parents to sign a permission slip opting their child in to receiving sex education at school [17, 19]. Additionally, the state legislature voted to implement new policies related to Student Health Advisory Committees (SHACs). SHACs play an integral role in sex education as they have the ability to work with community members to ensure the sex education provided represents the values of the community. Every school district is required to have a SHAC primarily consisting of parents who are not employed by the school district and who meet at least four times per academic year [17].

### 1.2 State board of education

The role of the SBOE is to select course materials and set health curriculum standards, the TEKS [20]. According to the TEKS, all Texas public schools must, at minimum, provide a health education course, which includes a chapter dedicated to abstinence-based sexual health information, to elementary (4^th^-5^th^ grade) and middle school students (7^th^-8^th^ grade) [21]. For high school students, the health course is optional, and does not have to be offered by public high schools [21]. In November 2020, the SBOE revised TEKS standards for the first time in over 20 years, resulting in the inclusion of topics such as contraception, sexually transmitted infections (STIs), and characteristics of healthy relationships for middle school students [21]. Conversely, the SBOE voted against requiring schools to discuss topics such as consent and human sexuality [22].

### 1.3 The current study

Sex education is instrumental in providing adolescents and young adults with the knowledge and skills to engage in healthy sexual decision making and relationships. High unintended pregnancy and STI/HIV rates among Texas youth highlight the need to examine current sex education policy and curriculum standards and emphasize sexual health prevention. In the United States, CSE has been proven to reduce STI/HIV and pregnancy rates among adolescents when compared to abstinence-based sex education [10]. Thus, the purpose of this study was to identify barriers and facilitators to implementing CSE at local and state levels in Texas.

## 2. Methods

### 2.1 Study design

For this study, we used a qualitative design to explore barriers and facilitators to implementing CSE policy and curriculum at local and state levels in Texas. A qualitative approach was a suitable method for this study as it allowed us to explore stakeholders’ beliefs and views about barriers to policy implementation in the state of Texas [23, 24]. Qualitative designs are particularly good, argues Sofaer [25] at “illuminating the experience and interpretation of events by actors with widely differing stakes and roles” (p.1101).

### 2.2 Participants

This study consisted of ten interviews with eleven key informants who were currently working or had previously worked in the field of sex education **(Table 1).** For one interview, two key informants were interviewed together, while the other nine interviews had one key informant. We recruited key informants who were involved in sex education policy and/or research or had contributed to the development or implementation of various forms of sex education curricula, in order to gain a comprehensive understanding of existing barriers and facilitators to facilitating policy or curriculum change at local and state levels. Additionally, key informants represented various regions, counties, and school districts in Texas.

**Table 1:** Key informants.

Key Informant Number	Profession
1	Assistant professor and sexuality education researcher
2	Assistant professor and health communication researcher
3	Policy director for statewide nonpartisan organization
4	Physician (obstetrician/gynecologist) and associate professor
5	Medical school director of community education
6	Policy director
7	Health and physical education supervisor
8	Communications strategist
9	Professor and researcher on prevention of pregnancy and STIs
10	Policy director and professor
11	Professor and school and community-based health researcher

### 2.3 Data collection

Semi-structured interviews ranging from 30–60 minutes were conducted. A semi-structured interview format was appropriate for this study as it facilitated data collection, while still allowing participants to freely discuss thoughts or ideas that might not have been considered by the research team [26]. Interview questions were drafted by the research team who specialize in policy and/or sexual and reproductive health to ensure content validity. The research team analyzed the Texas Education Code and TEKS Standards to develop interview questions aimed at identifying the individual, community, and societal-level factors that influence CSE policy and how these levels interact with each other [21]. Additionally, the interview questions were developed to apply to participants with various professions pertaining to sex education policy. Further, participant input contributed to the development of additional questions that were asked in future interviews. Key questions that guided the interviews are presented in **Table 2.**

**Table 2:** Key questions from interview guide.

1. Tell me about your experience of getting (or trying to get) comprehensive sex education on the policy agenda. 2. What are the biggest barriers that comprehensive sex education faces in moving forward? • Probe: How can these barriers be overcome? • Probe: Are there any upcoming windows of opportunity for prioritization 3. What has helped move comprehensive sex education up the policy agenda? 4. What do you think the impact of implementing comprehensive sex education would be on your city or state? 5. What role, if any, could you or your organization play in facilitating comprehensive sex education’s prioritization on the state policy agenda? 6. Can you identify any leaders or champions of comprehensive sex education? If so who? 7. The SBOE now requires parents to opt-in their children to sex education class. How do you think this new policy will impact Texas youth? * 8. For those who wish to change local and state sex education policy, what actions do you recommend they take? * 9. What changes would you make to current state sex education policy? * *Question added to Interview Guide after second interview

### 2.4 Study procedures

Initially, this study used purposive sampling to select participants based on the literature on CSE and personal knowledge of key actors in sex education policy. Purposive sampling was then supplemented by snowball sampling as initially selected participants were asked to provide referrals for additional potential participants. All selected participants were recruited via email from July 16, 2021 to October 25, 2021, and were provided information regarding the study. Once the participant agreed to the interview, they were provided with a written consent form that was signed before the interview. A total of ten one-time, semi-structured interviews were conducted with a total of eleven participants via Zoom after obtaining informed consent. By the fourth interview, five themes (three barriers and two facilitators) began to emerge and were solidified by the eighth interview. By the tenth interview, data saturation had been achieved. All ten interviews were conducted by one member of the research team (XX), who has training in qualitative research methods. To provide context for data analysis, field notes were written immediately after each interview to capture the interviewer’s thoughts, feelings, and perceptions.

### 2.5 Data analysis

All interviews were audio recorded and transcribed verbatim by the interviewer, and then independently coded using NVivo 12 software [27] by two coders (LH, SJ). Thematic analysis was used to identify barriers and facilitators to implementing comprehensive sex education. Each coder familiarized themselves with the data before identifying emerging themes in an effort to develop a codebook that would guide the data analysis process. Each coder independently analyzed the data and the two coders met intermittently throughout the coding process to discuss emerging themes and resolve any discrepancies. Once all interviews were coded, data from each coder was merged into one file to identify major themes and subthemes.

### 2.6 Researcher characteristics and reflexivity

It is important to acknowledge the research team members’ relationship to sex education policy. One member of the research team (XX) xxxxxxxxxxxxxxx where they are focused on xxxxxxxxxxxxxxxxxx. Another member (XX) is xxxxxxxxxxxxxxxxxx where they are focused on xxxxxxxxxxxxxxxx. One member of the research team (XX) is a xxxxxxxxxxxxxxx who focuses on xxxxxxxxxxxx.

### 2.7 Ethics approval

This study was performed in line with the principles of the Declaration of Helsinki. Approval was granted by the Duke University Institutional Review Board (Approved 07/02/2021; Protocol Number: 2021–0565)

## 3. Results

### 3.1 Barriers

We identified three key barriers to implementing CSE curricula and policy: 1) ideological opposition to CSE, 2) discrimination against lesbian, gay, bisexual, transgender, queer + (LGBTQ+) people, and 3) myths and misconceptions about CSE.

#### 3.1.1. Ideological opposition to CSE

Ideological opposition to CSE was found to be a major barrier to implementing CSE at the local and state level. Several participants stated that opposition from parents and advocacy groups was particularly problematic for school officials and policymakers. Two key informants (KIs) provided further insight into the conflict and difficulties faced by those who advocate for students to have an informative and inclusive sex education:

“*And they [opponents] can make life hell*, *for school officials*, *either an elected board member or an employee*, *like a superintendent of the district*. *They just get targeted by opponents who then misinterpret and misportray what’s taught in the classroom and try and scare people about what sex ed is being taught in their local school*. *And I think policymakers just don’t want the heartburn*. *And so*, *they in many cases end up not teaching sex ed at all*, *because they just*, *they don’t have to by state law*. *So*, *the easy thing to do is just not to teach it at all*.”—KI 8

“*But we’ve had pushback and we had actual, like protests and picketing of trainings, and school boards sort of boycotting, and people from outside the district attending school district meetings, to say that comprehensive sex ed was not age appropriate, that it was pornographic. That it’s not, you know, in the best interest of youth, and it’s not what school districts should be doing.”–*KI 9

Specifically, informants reported that resistance from parents was the most difficult barrier for school administrators. One interviewee (KI 4), a physician and associate professor who works closely with school administrators, stated, “*But also to administrators*, *they’re not so much afraid of the subject as they are parents*” and continued to cite a conversation they had with a school administrator who reportedly told her, “*I don’t want any more headaches*. *I don’t want to have parents calling me*”. A different KI said one reason for parental pushback is the belief that sex education should remain in the home:

“*The biggest barriers we saw, and we address this firsthand, were protests from others that don’t feel that way. That feel that sex ed is hurting young people, and that sexual health and sexual health discussion should be in the family, that families should be the primary sexual health educators for their youth. That negates the fact that a lot of kids don’t feel comfortable talking with their parents about these things, and sexual, gender and minority youth may not feel comfortable talking with their parents about these kinds of topics*.”—KI 9

#### 3.1.2. Discrimination against LGBTQ+ people

Implementing an LGBTQ+ inclusive sex education curriculum in public schools is a contentious subject in Texas, particularly among parents, advocacy groups, and the state legislature. For example, KI 3 said: *“And most of what they were opposing was the LGBTQ issue*. *That was that’s kind of become the big flashpoint*. *It’s almost like contraception is maybe like in the background a little bit now*. *And now the big thing is thing is*, *like*, *gender identity*.*”*

Although very few Texas youth receive a sex education that covers LGBTQ+ sexual health topics or issues, two informants reported that students continued to ask questions about LGBTQ+ sexual health and relationships in class:

“*The kids will talk about different kinds of relationships that are not represented in textbooks, and they’re seeing it on TV, they see it on their phones, they see it on this and it’s just ignored and overlooked in the educational realm.”—*KI 11

“*The school board doesn’t want us broaching the subject of homosexuality and*, *you know…all the letters*, *but the kids keep bringing it up to us*. *So*, *we keep telling them and SHAC [School Health Advisory Committee] every year*, *‘the kids are asking us*, *we need to be talking about this*.*’ And so far*, *they’ve kind of ignored us*, *but they won’t be able to ignore us much longer*.*”—*KI 4

Further, KI 4 stressed that providing LGBTQ+ inclusive sex education is important, because LGBTQ+ students are not being provided with sexual health information that could reduce their risk of STIs. She provided an example from her own experience as a sex education provider, *“… I was teaching STDs [sexually transmitted diseases]*, *and one girl said*, *‘Well*, *that’s okay*. *I can’t get them because I’m gay*.*’ And I said*, *‘No*, *if your partner’s infected*, *you can get them*.*’”*

Our study found that discrimination against LGBTQ+ individuals is perpetuated by misconceptions about LGBTQ+ inclusive curriculum. Informants reported that opponents of CSE will often falsify and sensationalize the LGBTQ+ sexual health information that would be provided to students in the classroom. KI 7, a textbook manufacturer, stated, *“… the way that they have opponents attack is they have to misportray what’s being taught in the classroom… that we’re indoctrinating students into the ‘homosexual lifestyle’*, *and we are confusing kids about gender*.*”* The same participant further elaborated on the misconceptions and general lack of understanding that elected officials have regarding the realities and hardship faced by LGBTQ+ youth:

“*…when proponents of that [comprehensive sex education] made the argument that teaching this kind of information helps lessen bullying, harassment, and suicidal ideation among LGBTQ youth, the board member who thought it was too controversial, actually questioned whether or not it was true that teaching this information would help prevent suicide among LGBTQ youth. Well, I’m gay. And let me tell you, it would. I mean, this is not something to be debated. And we know that this is the case.” -*KI 7

#### 3.1.3 Myths and misconceptions about CSE

Findings from our study showed that the sex education landscape is riddled with myths and misconceptions that prevent the implementation of sex education curriculum that is medically accurate, informative, and inclusive. One misconception, in particular, is that CSE encourages sexual activity among youth. KI 2 stated, *“If you do these things to make kids healthier*, *parents perceive and the community perceives that it’s a free pass to have sex*, *which is obviously not the case*.*”* Similarly, KI 3 argued, *“I think that there is a*, *you know*, *persistent belief that providing sex education to kids*, *sends them the message that it’s okay to have premarital sex*, *and will somehow make them more likely to have premarital sex*. *And the research tells us that that’s not true*. *But that’s the persistent belief*. *We see this in contraceptive access too*.*”*

The fear that sex education promotes sexual activity extends to the topic of consent, which is not included in the state-required health education course. Three study participants discussed why CSE opponents and a member of the SBOE did not want the topic of consent included in sex education curriculum:

“*And so for them [CSE opponents] teaching about consent apparently was like opening the door to sexual consent, it’s like really can go a complete misinterpretation of what people mean when they’re talking about affirmative and informed consent. But the board refused to adopt any standard that focused on affirmative and informed consent, largely for that reason.”- KI 8*

*“And so*, *one of the members said*, *he came out in the middle of the meeting*, *and he said that consent was a tool that pedophiles and like sex traffickers used to trick kids*. *And like*, *that was the moment where like*, *my jaw hit the floor*.*”–*KI 3

“*We had some concerns from one of the members of the board that teaching kids about consent encourages them to have sex when that is*, *you know*, *contrary to literally all research on the subject*. *And they pushed back really seriously*. *And we brought in like some human trafficking experts and those kinds of folks*, *because speaking about human trafficking tends to be a really robust conservative talking point in Texas*. *If you can get people to see it through a trafficking lens*, *they tend to be a little bit more amenable to like moving on an issue*. *And that didn’t work”*.–KI 6

Ultimately, the SBOE voted to exclude consent from the state-required health education curriculum. As an alternative, the SBOE shifted its focus to *“respecting the boundaries of other people”* (KI 3) because the SBOE *“felt much safer with the word ‘boundaries’ than the word consent”* (KI 5). KI 3 provided further insight into the shift from the word “consent” to “boundaries”:

“*So the old TEKS were like refusal, refusal, refusal, you have to refuse sex, just say no. And the new TEKS at least are like if somebody else says no, you have to respect that… It’s kind of like halfway to consent, right?”–*KI 3

### 3.2 Facilitators

We identified two key facilitators to creating policy and curriculum change: 1) sex education champions and 2) collaboration with community stakeholders.

#### 3.2.1. Champions

All research participants emphasized the importance of having sex education “champions” who advocate to expand sex education curriculum and policy to be more medically accurate, informative, and inclusive at local and state levels. In particular, support from healthcare professionals, parents, and youth was identified as essential to initiating sex education policy and curriculum change. For example, KI 4 shared how healthcare professionals played a role in making sex education curriculum medically accurate and more informative:

“*But there was a lot of misinformation. And they were several health professionals on the Student Health Advisory Committee that visited those classes. And some of them were really angry there. Some of them were really angry. But mostly they just wanted things to be more scientifically correct. And so they asked us to find a curriculum. And then they asked us to teach parts of that curriculum. They wanted anatomy and physiology, physiology of reproduction. They wanted contraception plus abstinence, and they wanted STI prevention and treatment.”–*KI 4

Two other participants discussed the importance of having healthcare professionals champion the expansion of sex education curriculum. KI 6 stated, *“But in addition to that*, *I would like to have folks hear from medical practitioners a little bit more*. *I think that our family planning doctors*, *and our pediatricians are the people who see the fallout of inaccurate sexual health teaching in our schools*. *And I think physicians also tend to be trusted voices in our communities*. *So*, *I think that kind of effort is important”*. Similarly, KI 11 stated, “*I think that helps to have physicians on board saying this is important*.”

Additionally, parents and youth play a critical role in advocating for CSE:

“*if you want to make change in your district, you need to get parents and community members on that SHAC, that are supportive of sexual health and comprehensive sex ed…”* They continued, *“…and you would want to make sure we always made sure that you had students and parents that were in favor of the particular program, whether that’s the evidence based program or this comprehensive program that you’re trying to get approved, and make sure that you have students from the district and parents from the district to stand up at the school board and say why this program is important, why it’s age appropriate, that students like it, that it has good outcomes.”–*KI9

In recent years, youth have also been using their voice to advocate for more informative and inclusive sex education curriculum by testifying in front of the SBOE:

*“There was not a single young person who showed up to testify at the State Board of Education, who was not speaking up in favor of sex education, consent, and inclusivity. Every single one of them that showed up, and knew exactly what they wanted in their education.”–*KI 3

“*And it’s just like*, *hours and hours*, *and hours of like smart*, *powerful young people being like*, *“I was literally taught nothing about my body*, *like*, *fuck you*.*”*–KI 5

#### 3.2.2. Collaboration with community stakeholders

Key informants emphasized the importance of collaborating with community stakeholders when facilitating conversations about curriculum with opponents of CSE. While a CSE curriculum that meets all twelve National Sexuality Education guidelines is ideal, it is not always possible to convince everyone to agree on every guideline. Thus, to move towards a medically accurate, informative, and inclusive sex education curriculum, collaboration is essential. For example, KI 7 said it is important to *“find that balance that everybody feels heard and respected”*. Two other KIs echoed the importance of striking a balance and collaborating with community stakeholders:

*“But, like, really getting buy in from the community, from the parents, from the medical from the youth serving professionals. And I think Austin ISD [Independent School District] did a good job of that. They did a lot more community outreach than they were remotely required to do by statute. They put out surveys of parents, they had a number of public hearings, a lot of opportunities for public comment, you know, they really tried to open up the process. As a people opposed to it would always, you know, if the process appears closed at all people that are opposed to what you’re doing will use that and make hay out of it*.”–KI 4

“*But you have to get the individuals to adopt the practice and understand the importance of the practice*. *So if you and you*, *you have to get people that are like minded to adopt the practice*. *So*, *the preacher of that church needs to understand that sex education in schools has these benefits*. *It doesn’t encourage sex*, *it actually discourages early sex it encourages safe sex practices*, *it decreases STI rates*, *it decreases teen pregnancy…”*–KI 2

Specifically, KIs 4 and 5, who work together, were successful in working closely with a local faith-based organization to implement a more informative sex education curriculum in their local school district. Curriculum topics were divided between a university medical school and the faith-based group. The medical school focused on science-based topics such as anatomy, contraception, and STIs, while the faith-based group focused on emotions and engaging in healthy relationships:

“*And so the superintendent said, you know, ‘we’re just not serving the kids, we’d love for them all to be abstinent, but if they aren’t, then we need to find a way to incorporate something for everybody.’ So, I knew we were probably going to have a bit of a fight on our hands, because the faith-based group was coming to the meeting as well. So, I came up with this plan where we could share the instruction.”*–KI 5

“*You know*, *there were some hiccups*, *but the people who were in charge of education at the faith-based group*, *were lovely people*, *and we really worked well with them*. *And they did have an abstinence only agenda*. *But they respected the fact that you know*, *we were trying to bring this the science component that the community asked for*.*”–*KI 4

They further discussed the importance and value of compromise:

*“…we spend a lot of time nurturing our relationship with the faith-based [organizations]. It could have been a bigger fight. And we chose not to fight. We chose to get along and we got an award for community involvement. And the three organizations- ourselves, the faith based, and the school system-got an award because we were working so well together.”–*KI 4

## 4. Discussion

This study adds to the current body of knowledge of CSE and Texas sex education policy by providing insight into barriers and facilitators to changing sex education policy and curriculum in Texas to be more inclusive, informative, and comprehensive. Our study identified three main barriers to policy change: ideological opposition to CSE, discrimination against LGBTQ+ people, and myths and misconceptions about CSE. The study also identified two key facilitators–sex education champions and collaborations with community stakeholders.

Findings from our study were similar to the few other studies that have specifically examined sex education in Texas. One major finding from our study was the fear of parental backlash faced by school administrators who wish to improve the sex education curriculum in their school. A similar observation was made in a study examining barriers faced by instructors in delivering sex education in West Texas [28]. Such findings highlight the importance of working closely with parents and other community stakeholders to ensure their voices felt heard throughout the developmental and implementation process.

Additionally, our study found that there are pervasive myths and misconceptions about sex education that fuel resistance to change. Similarly, a 2012 study examining sex education materials from 990 Texas school districts found that myths and misconceptions about the consequences of sexual activity were common in curricular materials [29]. In particular, the materials commonly used shame-based and scare tactics, which the researchers categorized into three types: “1) exaggerating negative consequences of sexual behavior; 2) demonizing sexually active youth; and 3) cultivating shame and guilt to discourage sexual activity” [29]. These findings show how sexual health misinformation in curricular materials is not a new phenomenon and serves as a major barrier in improving sex education curriculum. To dispel myths and break long-standing sexual health misconceptions, it is essential for public schools to provide students with medically accurate and informative sex education and for sex education advocates to engage with community stakeholders.

Our findings showed that community involvement, initiative and support are essential to implementing CSE. Similar findings have been demonstrated on an international level, where supportive school and community environments have been identified as facilitators of CSE implementation [30]. Concurrently, our study showed that a lack of community support for CSE was often attributed to LGBTQ+ discrimination. Community backlash is a major barrier for schools implementing LGBTQ+ inclusive protocols or sex education curriculum in the United States [31, 32]. More research is needed to understand how to engage in meaningful conversation and provide education about the relationship between LGBTQ+ inclusivity and health outcomes to CSE opponents.

### 4.1 Strengths and limitations

One strength of this study is the professional diversity of the key informants who work in various fields related to sex education. By interviewing a variety of experts, we were able to develop a more complete picture of the current state of sex education in Texas and of how policy change can occur. Another strength is the use of a semi-structured interview format, which allowed participants to share facts, personal experiences, and opinions on Texas sex education policy, while still answering interview questions developed to answer this study’s research question.

Along with its strengths, this study has two key limitations. First, the study sample mainly included viewpoints and insights from individuals who support implementing a medically accurate, informative, and, mostly, comprehensive sex education. It did not include perspectives of those who are against changing current school-based sex education curriculum or policy. Second, while we interviewed individuals who work with parents and students who advocate for sex education policy and curriculum change, we did not speak directly to the parents and students themselves.

### 4.2 Policy implications

Findings from this study provide insight into the opposition faced by sex education advocates, which often stems from myths and misperceptions of CSE content and the stigmatization of sexual and gender minoritized groups. Parents, youth, medical professionals, and academic researchers who support CSE are essential to dispelling sex education myths and misperceptions, and can move CSE up local and state policy agendas by advocating to their local schoolboard and state officials. Further, our findings highlight the importance of developing relationships and working closely with community stakeholders to gain a better understanding of overall community values. Working closely and compassionately with community stakeholders can increase local support for schools to implement a sex education curriculum that is more informative, accurate, and comprehensive than previously implemented curriculum.

Healthcare professionals and academic researchers are respected community members who can provide their medical knowledge, research, and work experience to dispel sex education myths, correct misunderstandings, and address concerns of sex education opponents. This study serves as a call to action for medical professionals and academic researchers to advocate for a medically accurate and more comprehensive school sex. Healthcare professionals and academic researchers can provide insight into important topics such as consent and LGBTQ+ sexual health to help reduce sexual assault, social stigma, mental health outcomes, and sexual health disparities.

### 4.3 Conclusion

While there are several obstacles to implementing CSE in Texas schools, measures can be taken to gradually expand sex education curriculum and policy to be more informative, inclusive, and effective. CSE advocates play a critical role in eliminating barriers by engaging with community stakeholders and getting involved with their local SHACs. Additionally, medical professionals and academic researchers who support CSE could play a key role in dispelling sex education myths and misconceptions. As Texas adolescents continues to be plagued by STIs, HIV, and unwanted pregnancy, it is important to shine light on current sex education practices, identify areas for improvement, and implement changes to policy that benefit the health and well-being of Texas youth.
