# Correction: Barriers and facilitators to implementing comprehensive sex education in Texas public schools: A qualitative study

**DOI:** 10.1371/journal.pone.0319359

**Published:** 2025-02-12

**Authors:** Lauren Holt, Sarah Janek, Gavin Yamey

In the ‘2.6 Researcher Characteristics and Reflexivity’ subsection of the Methods, "X’s" are used in place of author identifiers for the peer review process. This published version should include the author identifying information.

The correct section 2.6 is as follows: It is important to acknowledge the research team members’ relationship to sex education policy. One member of the research team (LH) is a PhD student at Duke University where she focuses on sexual health decision-making among adolescent and young adult women. She personally experienced sex education in a rural public school in Texas, inspiring the development of this study. Another member (SJ) is also a PhD student at Duke University, where she focuses on sexual health inequities and discrimination. She is an HIV/AIDS certified registered nurse, and experienced sex education at a small Catholic school in the Midwest region of the United States. One member of the research team (GY) is a physician who has conducted policy research on global reproductive health and written articles that advocated for CSE; he also used to write a weekly sexual health advice column for men who have sex with men in a young gay men’s magazine in London, England.
